# Of Energy and Entropy: The Ineluctable Impact of Aging in Old Age Dementia

**DOI:** 10.3390/ijms18122672

**Published:** 2017-12-09

**Authors:** Virginia Boccardi, Chiara Comanducci, Marta Baroni, Patrizia Mecocci

**Affiliations:** Institute of Gerontology and Geriatrics, Department of Medicine, University of Perugia, 06132 Perugia, Italy; virginia.boccardi@unipg.it (V.B.); chiaracomanducci@gmail.com (C.C.); martabaroni@libero.it (M.B.)

**Keywords:** dementia, elderly, entropy, energy, mitochondria, oxidative stress

## Abstract

Alzheimer’s disease (AD) represents the most common form of dementia among older age subjects, and despite decades of studies, the underlying mechanisms remain unresolved. The definition of AD has changed over the past 100 years, and while early-onset AD is commonly related to genetic mutations, late-onset AD is more likely due to a gradual accumulation of age-related modifications. “Normal brain aging” and AD may represent different pathways of successful or failed capability to adapt brain structures and cerebral functions. Cellular senescence and age-related changes (ARCs) affecting the brain may be considered as biologic manifestations of increasing entropy, a measure of disorder. Late-onset AD may be regarded as the final effect of a reduced energy production, due to exhausted mitochondria, and an increased entropy in the brain. This unique trajectory enables a bioenergetics-centered strategy targeting disease-stage specific profile of brain metabolism for disease prevention and treatment.

## 1. Introduction

Aging is the inevitable biological process that results in a progressive structural and functional decline from the cellular level to whole body function, which leads to reduced ability to adapt to environmental stress and changes. Along with aging, accumulation of cellular alterations leads to an increased risk of diseases and, ultimately, to death [1]. Thus, increasing knowledge on the molecular events that occur in the senescent cells is critical to understand the upstream processes of age-related diseases.

The aging population is growing worldwide, as well as age-related diseases, including neurodegenerative disorders, such as old age dementia. Cognitive decline represents a serious hindrance to achieving a long and healthy life. In fact, advancing age impacts negatively on cognitive abilities, and a mild impairment in brain functions is noticeable, even in physically healthy aged individuals, often heralding subsequent dementia [2]. Large population-based studies demonstrate an exponential increase in dementia incidence after 65 years of age, doubling roughly every five years, such that more than 50% of nonagenarians may be expected to suffer from dementia [3]. Alzheimer’s disease (AD) represents the most common form of dementia, accounting for 60 to 70% of all causes, and in 2016, the estimated annual incidence of AD in the oldest-old was approximately 37 new cases per 1000 persons [4,5]. Since the 1980s, people aged 85 and over are increasing dramatically, reaching 13% of the population over 65 [6]. They are defined as “oldest-old” and represent, today, the fastest growing age group in high-income countries. In 2010 in the United States, there were about 5–6 million people over 85, and this number is projected to quadruple in the mid-century [7]. Thus, the prevalence of dementia in this age group is not only very high, but continues to rise. Several age-specific studies have shown that, nowadays, about 45% of the oldest-old are affected by dementia, with 32% represented by AD [8].

But, “if we live long enough, will we all be demented?” wrote David A. Drachman in a letter published in 1994 [9]. Normal aging and AD might, in fact, represent a different pathway of successful or failed capability to adapt brain structures and cerebral functions. Thus, understanding their similarities and differences could be the key to solve the aging enigma.

This is a selective review focused on findings from studies investigating the impact of aging in dementia, and in particular, to try to find differences or similarities between normal aging and AD. A literature search in Pubmed, Medline, and Cochrane databases of all articles published with the medical subject heading keywords “aging”, “dementia”, “entropy”, “mitochondria”, “entropy and brain”, “brain aging and entropy”, and “Alzheimer’s disease” was carried out. The keywords were used in all possible combinations to obtain the maximal number of articles. All types of studies, from bench and animal models to clinical, were included. To answer our question, the review begins with a short overview of the ineluctable impact of aging in old age dementia. Then, by exploring brain aging at molecular level, we focus on studies that have examined the role of cellular senescence and ARC as biological manifestations of increased entropy. The review concludes with a general discussion that focuses on the mechanisms that underlie this unique trajectory, and implications for future research.

## 2. Peculiarity of AD in Old Age Subjects

Since Alois Alzheimer, at the beginning of the nineteenth century, first described the pathology and symptoms of a person with dementia whose brain contained neuritic plaques (NP) and neurofibrillary tangles (NFT), there have been many other classifications the disorder. Initially, and for some decades, AD was considered as a form of “presenile” dementia [9]. Old age individuals with cognitive impairment were not diagnosed as suffering from AD, but, usually, from “senile dementia”, even though their brains frequently contained neuritic plaques and neurofibrillary tangles. Thus, upon this classification, in the following decades, AD was considered a rather uncommon entity of presenile dementia, while senile dementia became progressively prevalent as life expectancy was increasing worldwide. But in 1976, Katzman, with a milestone editorial, stated: “Alzheimer’s disease and senile dementia are a single process and should, therefore, be considered a single disease” [10]. Since then, the definition changed to include, independently of age, all subjects with cognitive impairment and brain histopathological features of AD. After that, accumulation of epidemiologic data allowed researchers to identify two distinct AD populations: those with clearly recognizable genetic inheritance, and those without: the “early-onset AD” (EOAD) and the “late-onset AD” (LOAD). The first one is present at a younger age, and it is often recognized as due to autosomal dominant inheritance; the latter tends to manifest in the old age, and it is not associated with a deterministic gene mutation, although AD-affected relatives can be detected in the family history [11]. Genes that cause EOAD have been identified in *amyloid precursor protein (APP)* gene on chromosome 21, the *presenilin 1* gene on chromosome 14, and the *presenilin 2* gene on chromosome 1 [12]. Instead, many conditions have been proposed as risk factors of LOAD, most of them in common with cardiovascular diseases, what seems obvious considering that the oldest-old are often affected by multiple chronic pathologic conditions (multimorbidity) that can impact on the brain and cognitive functions. The high rates of incidence and prevalence of dementia in the oldest-old indicate that age, *per se*, represents the strongest risk factor for AD [13]. Low education and cognitive reserve, mid-life medical illnesses (diabetes, hypertriglyceridemia, hypertension), excessive alcohol intake or smoking, poor physical activity or diet, depression and chronic inflammatory status, have been associated with a high risk of LOAD [14,15]. Comparing EOAD with LOAD, traditional risk factors for AD lose their impact or have a paradoxical effect on the development of dementia. For example, the APO ε4 allele of apolipoprotein E, which encodes a protein detectable in plaques and neurofibrillary tangles, has been shown to have little effect on the risk of dementia in the oldest-old [13,14,16]. While high blood pressure increases the risk of AD in the middle age subjects, its effect in the older ones appears to be more protective, probably affecting cerebral hypoperfusion [17]. It has been shown that disease progression also differs with age, with a more severe and aggressive course in EOAD as compared to LOAD [18]. However, whether AD is a part of aging or whether aging is part of AD is still under debate, and the diversity in opinions need to be acknowledged.

During the past decade, a conceptual shift occurred in the field of AD considering the disease as a continuum. Solid evidence supports the conceptual frame that AD pathophysiological processes in the brain, such as deposition of amyloid and development of neurofibrillary tangles, can be identified years before the clinical onset of dementia. In this context, amyloid peptide 1–42, total Tau protein, and phosphorylated Tau in cerebrospinal fluid, positron-emission tomography (PET) with amyloid tracers, fluorodeoxyglucose-PET (FDG-PET) and structural brain magnetic resonance imaging (MRI) seem to be able to identify the pathological process of AD in the preclinical phase [19]. On this basis, the National Institute of Aging and the Alzheimer’s Association suggested dividing the pathophysiological markers into two major groups: (1) biomarkers of Aβ accumulation expressed as low cerebrospinal fluid (CSF) Aβ-42 and abnormal tracer retention on amyloid PET imaging, and (2) biomarkers of neuronal degeneration, represented by elevated CSF total Tau protein and phosphorylated Tau protein, decreased FDG uptake at temporal-parietal cortex on PET, atrophy of medial, basal and lateral temporal lobe, and medial and later parietal cortices on structural MRI [19,20]. However, while these biomarkers are useful and applicable in young and young-old subjects, in the oldest-old (over 85 years old of age), their exact role is not clear [19]. In fact, many clinicopathological studies showed a weak or lack of correlation between brain function and brain pathology in aged individuals. The major pathological hallmarks of AD, extracellular deposits of amyloid protein and intraneuronal NFT, are found with increasing frequency with advancing age [21], and postmortem studies have suggested that these markers are abundant in the oldest-old brains even without evident cognitive alterations. The fact that abnormal protein accumulation observed in AD is also present in the aging brain might suggest a causal relationship that, instead, is not as evident as commonly believed. Of note, the association between the pathological features of AD and dementia is stronger in younger persons than in the oldest-old [22], and patients with cognitive dysfunction may show a relatively mild neuropathology [23,24,25,26,27]. In the oldest-old, NFT preferentially involves the anterior part of the hippocampal CA1 field, with relative sparing of the other areas [28,29], neuritic plaques have a lower association with the development of AD [29], and only cortical atrophy seems significantly correlated with the diagnosis of dementia [20]. Also, many studies have found a positive correlation between cerebrovascular disease [22,30,31], cerebral amyloid angiopathy [32,33], and hippocampal sclerosis [34,35]—which are more common in the oldest old—and the development of cognitive decline. In a recent post-mortem study in subjects without cognitive impairment ranging from age 50 to 102 years, it was found that approximately half (47%) displayed Aβ deposition, and in 41% of the subjects over 80 years, a combination of Aβ, Tau, and α-synuclein was present, indicating that concomitant altered proteins are indeed common in the aged without a clinical impact [36]. Thus, accumulation of these proteins does not confer, alone, a major risk of dementia in the aged group. Authors concluded that these findings should be carefully considered while developing diagnostic biomarkers, particularly for identifying prodromal and preclinical AD in the elderly. Thus, other pathological alterations, such as soluble prefibrillar species of Aβ and Tau, probably better correlate to the cognitive dysfunction in AD [37]. Indeed, a more recent study aimed at relating cognitive abilities to the degree of brain atrophy, CSF biomarker levels and neuropathology in a large cohort of aged men, found only a weak correlation between cognitive performances and medial temporal atrophy, while neuroradiological, biochemical, and neuropathological measures did not correlate with each other. In light of such evidence, authors concluded that AD biomarkers seem to be less informative in subjects with advanced age [38].

Collectively, these findings suggest that while amyloid can be considered as a “hallmark” of AD, its relationship to cognitive decline is not so consistent in the elderly, and it might not be, at this age, the optimal target for treatment in LOAD. It seems rather that in old age subjects, amyloid accumulation, as well as aggregation of other misfolded proteins, enters into the process of aging but it is still unclear, or likely underestimated, what triggers neuronal death and subsequent clinical manifestations. Most of the pharmacological products used currently in clinical trials and focusing on disease modification of AD are based on the amyloid hypothesis, and target different aspects of amyloid metabolism. However, the recent failures of drugs targeting amyloid pathways have raised questions not only about this approach, but also on the validity of the amyloid hypothesis itself. The considerable discrepancy between pathology and dementia in the oldest-old has focused attention on the greater importance of neuronal loss, rather than the accumulation of abnormal protein deposits in causing cognitive impairment. For decades, it has been a commonly held notion that widespread neuron death in the neocortex and hippocampus is an inevitable concomitant of brain aging. However, recent quantitative studies suggest that neuron death is not widespread in normal aging [39], suggesting that age-related cognitive impairment and clinically evident dementia are not part of a continuum, and the individual phenotype does not mirror the histopathological alterations in the brain. The lack of association between amyloid senile plaques (SP) and NFT and cognitive functions has led to the construct of the “cognitive reserve”, the hypothesized capacity of the mature adult brain to resist the effects of diseases or injuries that would otherwise cause dementia [40]. According to this view, elderly individuals with a high level of cognitive reserve may remain dementia-free in spite of neuropathological changes. Dementia could result from the continued accumulation of damages due to potentially preventable age-related risk factors [41], eventually surpassing a threshold after which protective and compensatory mechanisms are insufficient to guarantee a healthy cognitive status. Since aging is an inevitable process, managing modifiable risk factors could, at least partially, prevent or delay some of the devastating aspects of dementia in the old-age. Achieving exceptional longevity by slowing the aging process and reducing the impact of age-related diseases, may offer a winning approach. Thus, the control of different risk factors, some of them potentially modifiable, will determine the individual’s probability of remaining in good physical and cognitive health.

## 3. Cellular Senescence, Age-Related Changes, and Brain Aging

Dementia is an umbrella term used to describe diseases secondary to neuronal death and dysfunction. The fact that both neurofibrillary tangles and neuritic components of plaques of subjects affected by AD occurred with immunoreactivity against senescent biomarkers (and in particular p16^Ink4a^) [42], suggested the potentially central role of “cellular senescence” in this disease. Cellular senescence was originally identified in “permanent” cell cycle arrest, due to the limited replicative capacity of normal human diploid fibroblasts in culture. Recently, three distinct origins of senescent cells have been described. The first is cells that have reached their replicative limits and have lost their ability to proliferate further, termed “replicative senescence”. The second is “cellular senescence” induced by a variety of stimuli, including DNA damage, oxidative stress, chromatin distortion, and replicative stress, able to induce the cell cycle arrest before cells lose their proliferative capacity. The third is “non replicative senescence” in terminally differentiated non-proliferative cells (such as neurons), which acquire key features of senescent cells [43]. Senescence is a process characterized by alterations in cell morphology, metabolism, gene expression, and epigenetic regulation [44]. In particular, several studies have identified phenotypical molecular changes, such as altered cell morphology, secretion of inflammatory cytokines, bioactive peptides, and growth factors, proteases [45,46], that were called senescence-associated secretory phenotype (SASP). This phenotype, along with an increased expression of the cell cycle regulating protein p16^Ink4a^, β-galactosidase activity, and heterochromatin foci, is the principal marker of cellular senescence [45,46,47,48]. The senescent cells also release reactive oxygen species (ROS), which promote DNA damage and, in turn, increased levels of ROS lead to progressive loss of tissue homeostasis. Recent studies identified markers of senescence in mouse neurons after exposure to oxidative and metabolic stress [49]. Neurons accumulate lipofuscin in the cytoplasm and extralysosomal waste materials, such as protein aggregates [44]. Microglia—the principal effector of the innate immune system in cerebral tissue, playing an essential role in maintaining the plasticity of the neuronal circuits, protecting and remodeling the synapses—exhibit pro-inflammatory phenotypes during aging, with an increased expression of pro-inflammatory cytokines [50]. Finally, astrocytes, although numerically unaltered [51,52], present structural abnormalities characterized by an increasing amount of lipofuscin, glial fibrillary acidic protein, and vimentin filaments in the cytoplasm [46].

**Figure 1:**
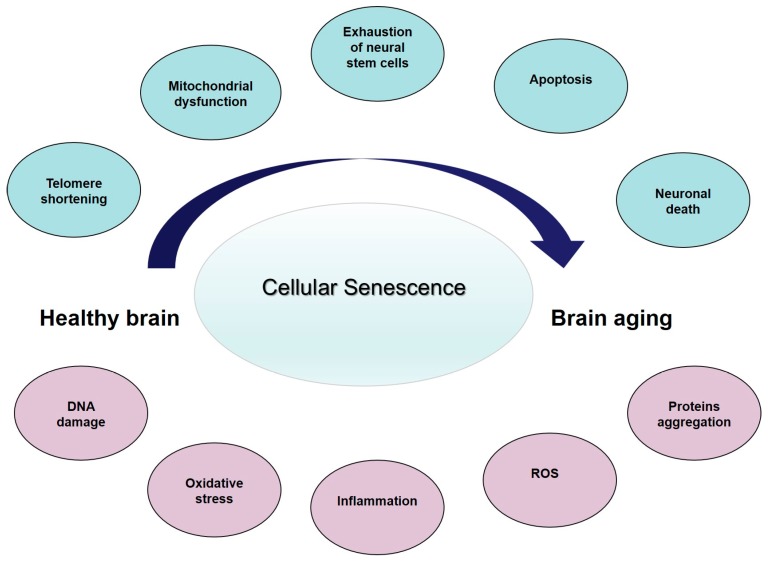
From healthy brain to brain aging: insights into age-related changes (ARCs). Some proposed ARCs with cellular senescence as the central contributing factors to age-associated brain tissue dysfunction. Blue circles represent the main changes while in pink represent the consequences and downstream events. ROS: reactive oxygen species.

Cellular senescence is a major contributing factor to age-associated cerebral dysfunction (reviewed in [46]), and represents the core feature of the so-called age-related changes (ARCs) (Figure 1). Collectively, the ARCs arise from intrinsic and extrinsic causes [53]. Intrinsic ARCs are those that result from programmed processes, while extrinsic ARCs are the result of experiential wear and tear and of randomly occurring, or stochastic, damaging events during life. Along with aging, and secondary to these cellular events, the structure and function of the brain progressively change. It has been consistently found that brain volume and/or weight declines with age at a rate of 5% per decade after the age of 40, with an increase in loss over age 70 [54]. A study looking at cortical thickness and white matter hyperintensity volume in people aged 50 to 81 years, pre-screened for dementia and depression, found an association between increasing age, reduction in prefrontal cortical thickness, increased subcortical white matter lesions, and more evident perseverative behavior as expressed by decreased executive function [55]. Neurons progressively reduce in the substantia nigra, mesial temporal region, and hippocampus. At the same time, the number of NP and NFT increase [56]. Thus, it possible to hypothesize that the brain changes affecting cognition and behavior start at the molecular level, through tissue aging and finally to the organ changes. Collectively, these data suggest that neurons are subject to cellular senescence as a function of accumulated DNA damage, oxidative stress, or inflammation, which increase during brain aging and as well as in neurodegenerative diseases, such as AD (reviewed in [46]). A literature analysis of cellular changes related to senescence in brain aging and AD [46] suggest that the differences between the two conditions are quantitative, rather than qualitative. The presence of senescent cells in the brain may contribute to the pathogenesis of AD, and may represent a link between the aging process and disease progression laying the basis for innovative anti-AD therapeutic strategies.

## 4. Aging and Dementia: An Entropic Point of View

The effects of ARCs in the brain may be seen as the biological manifestation of increasing entropy. Biological aging represents the biomedical counterpart of the irreversible increasing entropy of the system, where ARCs are the specific molecular components. In detail, the several biological and non-biological theories and hypotheses of aging can be classified into two principal categories. The first one considers aging as a programmed process driven by different genes. According to this view, cellular health is regulated at various points in the cell: from the nucleus through chromosome structure organization, transcriptional regulation, and nuclear trafficking, ranging outward to protein translation, proteostasis and autophagy, maintenance of cytoskeletal integrity and, finally, maintenance of the extracellular matrix and extracellular signaling. Each regulatory system receives information from the others, resulting in an intricate interplay of regulation controlling cellular life and aging [57]. Non-programmed theories, also known as non-adaptive, consider that aging does not have a “selectable” evolutionary purpose. These theories contend that aging is a sort of inevitable adverse side-effect of vital biological function, or that organisms do not have an evolutionary need to live after reproduction, and therefore, did not develop repair capabilities. Several theories on the mechanisms of aging are based on the assumption that during life, a continuous “wear and tear” of the organism takes place. For example, an increased metabolic activity would result in accelerated cellular aging. In this context, among the non-programmed theories, the most logical and attractive is the “entropy theory of aging”, a sort of wear and tear concept. It considers aging as a sequence of discontinuous steps, leading the cell to various stages characterized by a decrease in energy production and an increase of internal entropy, associated with a critical threshold of error accumulation [58]. The progressive deterioration is the biological expression of the general second principle of thermodynamics, also called the law of entropy, which states that energy processes move from order to disorder, and that as energy is transferred or transformed, more of it is wasted. Dissipative systems, such as living cells, are open systems, and they are moved away from equilibrium by the fluxes of material and energy across their boundary, and maintain their structure by continuous exchanges of energy and matter [59]. Entropy, “transformation” in ancient Greek, is the measure of disorder, and its production is directly related to general metabolism because the core of energy in the cell is linked to enzymatic reactions either in catabolism or anabolism [60]. Prigogine and Denbeigh defined the internal entropy in open systems as Ds: Ds1 + Dse. In other words, the total entropy change (Ds) is the sum between the internal entropy generated by all the biochemical reaction (Ds1) and the entropy which is exchanged with the environment (Dse) [61]. Clausius instead defined entropy mathematically as ΔS: $\frac{\Delta Q}{T}$. Where ΔS is entropy, Δ*Q* is the reversible change in the heat content of a body, and T is temperature. He stated that “it is impossible for any self-operating device to take heat continuously from a reservoir at one temperature and deliver it to a reservoir at a higher temperature”. In other words, heat flows on its own from high temperature to low temperature [62]. Aoki [63], estimated that metabolism accounts for 99% of total entropy production. Of the total entropy finally produced from metabolites entering catabolism, about 40% is transformed into the energy-rich phosphodiester bonds by ATP and the reducing NADPH [64]. This free energy is used by the cell to perform all the energy-requiring functions, like maintaining its structure by continuous synthesis of cell components, by housekeeping activity, and by using energy for movements and interactions with the environment and other surrounding cells [65].

## 5. Bioenergetics in Brain Aging and AD

Energy metabolism of the brain accounts for about 20% of the total body basal oxygen consumption [66,67]. This energy is derived from the aerobic oxidation of glucose. Lower brain glucose metabolism is present before the onset of clinically-measurable cognitive decline. In addition, brain aging is associated with a decreased glucose uptake and with a reduced glucose transporter expression. Glucose enters neurons from blood vessels via GLUT-3 and GLUT-4, the insulin-sensitive transporters. Several studies have shown that in brain aging, the expression of these transporters is dramatically reduced, while the expression of GLUT-1, present in the endothelial cells, slightly decreases [68]. The brain utilizes about 25% of the body’s total glucose, requiring 120–130 g glucose every day [69]. Emerging evidence from in vitro and animal studies suggest that brain hypometabolism may precede and contribute to the neuropathological cascade, leading to the cognitive decline in AD. The reason for brain hypometabolism is unclear, but may include defects in glucose transport and mitochondrial function [70]. Mitochondria are the core system of cellular energy supply, and they are particularly important because 90% of the glucose (the primary source of brain energy) is oxidized to CO_2_ [71,72]. The energy generated in this process is utilized to maintain neurotransmission and neuronal potential, to maintain neuronal structure, and to guarantee all structural and functional anabolic and catabolic processes [72]. Thus, any alterations to neuronal glucose metabolism, largely supported by mitochondria, would affect neuronal function and ultimately cognition. A recent review [71] on proteomic studies of mouse brain mitochondria during aging identified functional deficits and alteration in the expression of several proteins. In particular, aging brains display a reduced expression of electron transfer proteins of the oxidative phosphorylation (OXPHOS) system, especially components of the complex I–III–IV–V [72,73]. This results in a reduction of overall energy metabolism, with decreased activity of the more efficient mitochondrial OXPHOS system, and a shift towards the less efficient glycolytic pathway for energy production. Furthermore, with aging, mitochondria tend to produce more reactive oxygen species (ROS), a by-product of OXPHOS activity, that in turn can damage all biomolecules inside mitochondria and, more generally, in the cell [71]. To guarantee ATP production and prevent membrane depolarization, mitochondria undergo structural changes through processes known as fusion and fission. These modifications may act as a compensatory mechanism to preserve function and prevent the accumulation of dysfunctional mitochondria [65,74]. Importantly, fusion and fission also increase with age to maintain the overall morphology of the mitochondrial population, to guarantee the same energy level, and avoid the selective elimination of damaged mitochondria by autophagy (termed mitophagy) [71]. Alterations of these dynamics can cause mitochondrial dysfunction and cellular senescence.

A progressive age-related accumulation of oxidative damage to DNA in the human brain and mitochondrial DNA have been found [75], suggesting that such damage may contribute to the age-dependent increase in the incidence of neurodegenerative diseases. Mitochondrial DNA is particularly sensitive to oxidative damage, and we showed that, compared to healthy aged subjects, there is increased oxidative damage to DNA in the AD brain, as measured by the oxidized nucleoside 8-hydroxy-2′-deoxyguanosine (OH8dG) [76]. The interaction between oxidative stress and mitochondrial dysfunction likely forms a vicious downward spiral that amplifies the alterations observed in AD. With these observations, the “mitochondrial cascade hypothesis” has been proposed to explain AD pathogenesis [77], suggesting that every single person has a genetically determined mitochondrial endowment that, together with environmental factors, determine mitochondrial aging, reducing energy production, decline in physiological functions, and increased disorder of the system (i.e., increased entropy). Thus, the “mitochondrial cascade hypothesis” places mitochondrial dysfunction as the leading factor in the LOAD pathology cascade, underlying the individual genetic background able to regulate, since birth, its mitochondrial function and sustainability. For this reason, the rate at which age-related mitochondrial dysfunction proceeds differs among individuals. When the mitochondrial function declines and falls below a critical threshold, AD-typical dysfunction at the cellular level may ensue, including β-amyloid production, Tau phosphorylation, synaptic degeneration, and oxidative stress [78]. In this context, increased amyloid may simply represent a downstream event, the expression of a cooling system. This creates a vicious cycle in which excessive Aβ accumulation and sustained mitochondrial dysfunction synergizes to activate a cascade of neurodegenerative pathways. The progressive reduction of capacity to produce, store, and maintain a high energy level, which is the primary role of mitochondria in the cells, reflects increased entropy that progressively leads the organism from function to dysfunction, and then from life to death, the expression of the maximal entropic status. “If we live long enough, will we all be demented?” [9]. The proposed view may provide an affirmative answer, however, upon current evidence the response could be “maybe”. More biological and clinical studies, as well as clinical trials on elderly populations, are needed to support the bioenergetics view of AD. A threshold for normal cognitive aging and the more catastrophic AD is impossible to mark, considering that brain aging is heterogeneous, and where brain and cognitive reserve play an important role inter-individually.

## 6. Final Remarks and Perspective

Cellular senescence—the main contributing factor to age-associated brain tissue dysfunction—may represent the core feature of ARCs (Figure 1), leading to an overall reduction in brain structure and function. All together, these changes can be considered as the result of an “energy failure” which leads neurons and other brain cells to a progressive decline, which ultimately limits the functional brain capacity. Since the brain is not a closed system, this decline may be different among individuals. Thus, physiological variations in susceptibility to ARCs may determine why some individuals maintain cognitive competencies in advanced age, while others develop LOAD. So we could hypothesize that the protein misfolding and aggregation we observed in aging brain, and then in the late-onset AD, is the final effect of reduced energy production, due to exhausted mitochondria, and an increased entropy in the brain. The concept of the late-onset AD because of increasing entropy—with an accelerated, catastrophic decline when homeostatic mechanisms fail—suggests that strategies designed to modify the course should precede the shift from the modest decline in normal aging to the rapid tissue loss in AD. Thus, it seems critical to reconsider late-onset AD as a complex condition with a prolonged trajectory of changes in the brain, characterized by a progressively reduced metabolism and impaired bioenergetics. These changes start many years before the clinical onset of dementia, which supports incapacity of a biological system to maintain the molecular order that guarantees life, thanks to a steadily high energetic support. Considering the role of mitochondria in cellular bioenergetics, the decline in mitochondrial function probably represents the crucial factor. This unique trajectory enables a bioenergetic-centric strategy that targets a disease stage-specific profile of brain metabolism for disease prevention and treatment: it depends on modifying as many ARCs as possible to delay and slow the increasing disorder due to entropy and avoid loss of brain function, and increased neural vulnerability, as long as possible. Some strategies for reducing ARCs and conserving neural integrity may already exist (healthy lifestyle and diet, limiting ROS and inflammation, avowing obesity and other cardiovascular risk factors, and improving physical exercise) whereas others remain to be discovered.
